# Encapsulating Peritoneal Sclerosis in a kidney transplant recipient - Case Report

**DOI:** 10.1590/2175-8239-JBN-2019-0193

**Published:** 2020-05-08

**Authors:** Bruno Henrique Dantas Ribeiro, Vanessa Suemi Takenaka, Felipe Sbrolini Borges, Thales Franco de Andrade, Sibele Braga Lessa, Jorge Marcelo Padilla Mancero, Irene L. Noronha, André Ibrahim David

**Affiliations:** 1 Hospital Beneficência Portuguesa de São Paulo, Equipe de Nefrologia e Transplante de Rim e Rim-Pâncreas, São Paulo, SP, Brasil.; 2 Hospital Beneficência Portuguesa de São Paulo, Instituto de Gastrocirurgia Avançada, Programa de Cirurgia Geral e Aparelho Digestivo, São Paulo, SP, Brasil.; 3 Hospital Beneficência Portuguesa de São Paulo, Instituto de Gastrocirurgia Avançada, Programa de Transplante de Fígado, São Paulo, SP, Brasil.; 4 Universidade de São Paulo, Faculdade de Medicina, Laboratório de Nefrologia Celular, Genética e Molecular, São Paulo, SP, Brasil.

**Keywords:** Peritonitis, Peritoneal Dialysis, Organ Transplantation, Kidney Transplantation, Peritoneal Fibrosis, Peritonite, Diálise Peritoneal, Transplante de Órgãos, Transplante de Rim, Fibrose Peritoneal

## Abstract

Encapsulating Peritoneal Sclerosis (EPS) is a severe and rare condition frequently associated with peritoneal dialysis, characterized by bowel obstruction, with lethal consequences in 20% of the patients. The disease presents as a mass of fibrous tissue encapsulating visceral organs that may potentially compromise digestive tract function. This report describes the case of a patient under peritoneal dialysis (PD) due to chronic kidney disease secondary to focal segmental glomerulosclerosis diagnosed with EPS. The patient had undergone two living-donor kidney transplant procedures. Surgical techniques and clinical measures employed to unravel bowel obstruction are described, which have been shown to ameliorate EPS secondary complications. Parenteral nutrition has significantly contributed to afford adequate nutrition, improving tissue healing as well as serum protein levels, vitamins and electrolytes. Therapy with tamoxifen and sodium thiosulfate effectively delayed the development of EPS.

## Resumo

A peritonite esclerosante encapsulante (PEE) é uma condição rara e grave, frequentemente associada à diálise peritoneal, caracterizada por obstrução intestinal, que pode ter uma evolução fatal em 20% dos pacientes. Apresenta-se como uma massa de tecido fibroso, recobrindo os tecidos viscerais, e pode comprometer o funcionamento fisiológico de todo o aparelho digestivo. O presente estudo relata um caso de PEE decorrente de diálise peritoneal (DP) devido à insuficiência renal por glomeruloesclerose focal e segmentar. No caso relatado, a paciente foi submetida a DP e passou por dois transplantes renais intervivos. São descritas as técnicas cirúrgicas e as medidas clínicas adotadas, que se revelaram úteis na resolução da obstrução intestinal, com melhora dos efeitos secundários da PEE. A dieta parenteral mostrou ser um importante fator para a manutenção do aporte nutricional, auxiliando na cicatrização e no nível sérico de proteínas, vitaminas e eletrólitos. A terapia com tamoxifeno e a administração de hipossulfito de sódio foram eficientes para retardar o avanço da PEE.

## Introduction

Encapsulating Peritoneal Sclerosis (EPS) is a severe complication seen in patients on peritoneal dialysis (PD). It is a rare adverse event, with incidence ranging between 0.9% and 7.3%.^1^ The occurrence of EPS has been associated with time of exposure to dialysis solution. As many as 19.4% of the patients on dialysis for longer than eight years develop EPS,^2^ a condition with morbidity and mortality ranging between 25% and 55%.^3^

EPS is a chronic inflammatory condition of unknown etiology that manifests clinically and sub-clinically. The peritoneum changes into diffuse fibrous tissue and forms cocoons that encapsulate the viscera, resulting in fibrotic visceral constriction. Bowel motility is consequently impaired and severe complications such as bowel necrosis, enterocutaneous fistula, EPSsis, and death may occur.^1^

The main risk factors for EPS are duration of PD and repeated episodes of peritonitis.^3^^,^ 4 Nonetheless, reports have indicated links with beta blocker therapy and prescription of hyperosmolar solutions.^4^ Increases in the incidence of EPS in the first years after kidney transplantation have been recently reported.^5^

The diagnosis of EPS is based on clinical signs of bowel obstruction and evidence gathered through imaging or surgery findings of a thickened peritoneal membrane resulting in the encapsulation of the bowel. Early confirmation helps to prevent bowel complications, which may develop even after patients are no longer on PD.^6^^,^ 7

Most of the few reports published in the literature about surgery for EPS have cited enterectomy, lysis of bowel adhesions, or peritonectomy.^8^

## Objective

This paper reports the case of a patient on PD previously submitted to two kidney transplant procedures diagnosed with EPS based on signs of bowel obstruction.

## Case Report

A 25-year-old female was diagnosed with focal segmental glomerulosclerosis when she was two years old. She was treated with steroids, but progressed to peritoneal dialysis (PD) at the age of three.

Seven months later, when she was aged four, the patient was submitted to a first kidney transplantation with living related donor (mother), at another Transplantation Center. The immunosuppressive regimen consisted of cyclosporine, azathioprine, and prednisone. Due to low adherence to the immunosuppressive medications, she developed chronic rejection at the age of 16 - twelve years after transplantation - and returned to PD.

At the age of 20, the patient underwent a second kidney transplantation with living related donor (father). She developed biopsy-proven humoral rejection, and the kidney allograft had to be removed. The patient restarted PD. Three years and three episodes of peritonitis later, notwithstanding the fact that her peritoneal fluid effluent was cloudy and sandy, she refused to switch to hemodialysis (HD).

At the age of 24, the patient started losing weight associated with diffuse abdominal pain, nausea, and vomiting. She came to the Hospital Beneficência Portuguesa de São Paulo (BP) and was hospitalized with bowel obstruction. Laparoscopic biopsy of the peritoneum showed she had peritoneal sclerosis. She was suspected for EPS and prescribed therapy with prednisone 40 mg/day and tamoxifen 40 mg/day. She was switched from PD to HD.

Six months later and aged 25, the patient was hospitalized again at BP for bowel obstruction caused by EPS. She was started on intravenous 25% sodium thiosulfate (80 mL diluted in 200 mL of 0.9% saline solution) three times a week administered in the last hour of the hemodialysis session. The patient had a good clinical response to this treatment, recovering the ability to defecate. She was discharged without gastrointestinal complaints. The patient went back to her hometown. However, the treatment was interrupted due to the unavailability of this medication in her hometown. The symptoms returned.

**Figure 1:**
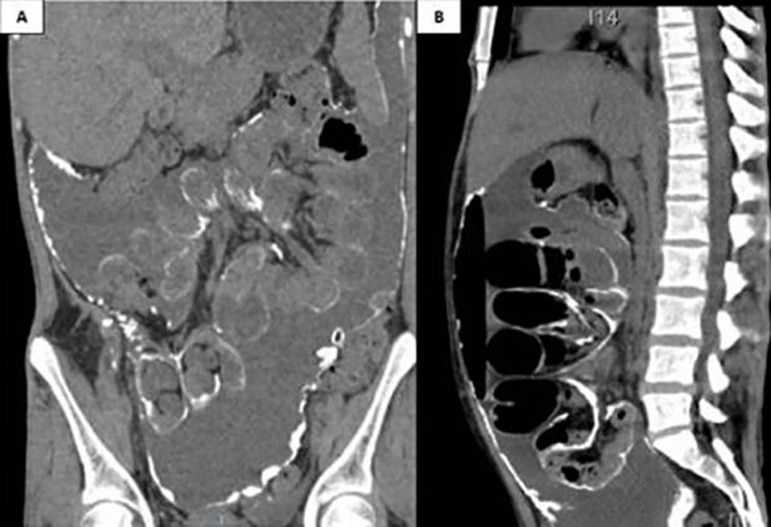
Computed tomography scans of the abdomen and pelvis; coronal view **A)** and sagittal view **(B)** showing a thickened and calcified parietal and visceral peritoneum, swollen bowel loops, pneumoperitoneum, ascites, and deposition of material in the peritoneal flexure.

**Figure 2:**
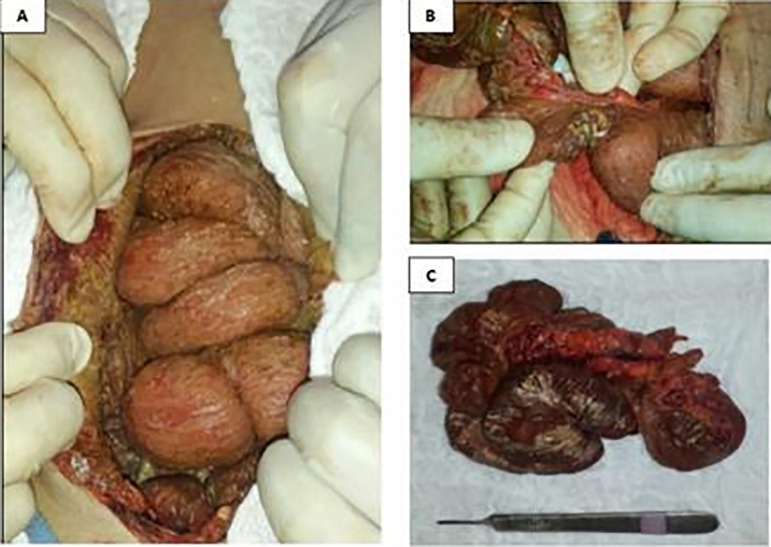
Anomalies found during surgery. **(A):** Diffuse calcifications in the bowel loops; **(B):** Stenosis of the bowel lumen and terminal ileum; **(C):** Resected segment of the ileum and right colon with fibrosis and stenosis of the terminal ileum.

Five months later, she was hospitalized again at BP with bowel obstruction. Computed tomography (CT) scans revealed diffuse thickening of the parietal and visceral peritoneum with extensive calcification involving the mesentery and bowel loops. She was also diagnosed with moderate pneumoperitoneum and ascites (Figure 1). Video laparoscopy showed large amounts of purulent fluid and stenosis in the terminal ileum and cecum. The patient underwent open surgery, which found gross calcification in the intestinal cavity and altered anatomy of the small and large bowels (Figures 2A e 2B), in addition to confirming the existence of bowel obstruction. The terminal ileum with stenosis was removed (Figure 2C), the ascending colon sutured, and a terminal ileostomy performed. The abdominal cavity was washed and drained. The anatomo-pathologic findings of the surgery specimen indicated the patient had fibrino-leukocytic chronic serositis coupled with fibrosis and hyalinization, associated with multiple sites with dystrophic calcification and an ileocecal valve with lipomatous hypertrophy.

The patient was kept on parenteral nutrition to improve nutrient uptake. She had a good clinical outcome her condition improved. The ileostomy was functional, and the patient was later switched to oral oral diet.

Three months later, the patient had a hemorrhagic stroke and died.

## Discussion

The patient described in this case report had long term chronic kidney disease. Until she developed EPS after being on PD for more than eight years and being the recipient of two kidney transplants. The etiology of EPS is unclear. In addition to infection (peritonitis), it is possible that the peritoneum had been preconditioned by PD solutions, which cause permanent progressive inflammation. In this context, it has been described the important role of transforming growth factor-ß mediating fibrosis progression and bowel loop encapsulation. In kidney transplant patients, the surgical trauma associated of the transplantation procedure associated with the inflammation caused by PD solutions may have contributed to the development of peritoneal fibrosis. Additionally, the use of calcineurin inhibitors, which induce the expression of TGF-ß, have possibly contributed to the development of fibrosis.^9^^,^ 10

The early clinical features of EPS may be unspecific, the most common of which being abdominal pain, weight loss, nausea, and vomiting.^4^ Early diagnosis and intervention may delay the progression of the disease and its complications.

The management of EPS is complex. In patients with abdominal complications from EPS, surgery is required to treat bowel obstruction, enterocutaneous fistulae, and bowel necrosis. Abdominal lavage and removal of intraperitoneal calcifications may help to combat infection.^8^ The patient described in this case was submitted to a thorough debridement of excess fibrous tissue encapsulating small bowel loops and parietal and visceral peritoneum. Along with surgery, dietary support is of crucial importance and must be started early on. Oral and enteral supplements may be used, while parenteral nutrition is also an option.^11^^,^ 12 Proper nutrient intake is important for the healing process.^11^

There is no consensus on drug therapy for EPS. Immunosuppression with steroids, azathioprine, or mycophenolate mofetil after transplantation or as specific therapy may produce good results.^13^ In case of failure of these treatments, Tamoxifen may be considered an option, considering previous reports in the literature describing regression of retroperitoneal fibrosis and EPS associated with Tamoxifen adminstration.^14^^,^ 15 ^,^ 16

Tamoxifen, a selective estrogen receptor modulator, may be a therapeutic option to block processes connected with fibrosis. Tamoxifen has been used to combat fibrosis in a number of cases of idiopathic retroperitoneal fibrosis, EPS, and fibrosclerotic disorders such as desmoid tumors and fibrosing mediastinitis.^16^ The drug’s antifibrotic effects have also been described in other disease models associated with fibrosis. In addition to clinical evidence of fibrosis regression, in vitro studies have linked tamoxifen to antifibrotic effects.^14^ Tamoxifen suppresses the transcription and synthesis of collagen, decreases the expression of TGF-ß, and inhibits fibroblast proliferation.^14^^,^ 16

Our patient suffered with severe calcification involving even the peritoneal membrane, indicative of a state of calciphylaxis. Risk factors for calciphylaxis include calcium and phosphorus metabolism dysregulation stemmed from chronic kidney disease, prolonged periods on PD, and long term administration of high-dose calcium-based phosphate binder therapy.^17^

Intravenous or intraperitoneal sodium thiosulfate has been prescribed to treat calciphylaxis.^18^^,^ 19 Sodium thiosulfate is a potent antioxidant and improves calcium deposit solubility. Intravenous dosage for adults ranges between 5 and 75 g after or during hemodialysis. The most commonly prescribed dose is 25 g after each dialysis session.^20^ Infusion times vary between 30 and 60 minutes. Although mostly well tolerated, the drug’s adverse effects include nausea, vomiting, and metabolic acidosis.

In conclusion, this case report stresses the relevance of surgery in the treatment of complications arising from EPS, to either rid patients of bowel obstruction - a known complication of this condition, or to wash the abdominal cavity and remove calcifications. Adjuvant therapy with tamoxifen and sodium thiosulfate may also be used to control fibrinogenesis and calciphylaxis, respectively.

## References

[1] Stuart S, Booth TC, Cash CJC, Hameeduddin A, Goode JA, Harvey C (2009). Complications of continuous ambulatory peritoneal dialysis. Radiographics.

[2] Kawanishi H, Kawaguchi Y, Fukui H, Imada A, Kubo H, Kin M (2004). Encapsulated peritoneal sclerosis in Japan: a prospective, controlled multicenter study. Am J Kidney Dis.

[3] Latus J, Ulmer C, Fritz P, Rettenmaier B, Biegger D, Lang T (2013). Encapsulating peritoneal sclerosis: a rare, serious but potenially curable complication of peritoneal dialysis- experience of referral centre in Germany. Nephrol Dial Transplant.

[4] Habib AM, Preston E, Davenport A (2010). Risk factors for developing encapsulating peritoneal sclerosis in the icodextrin era of peritoneal dialysis prescription. Nephrol Dial Transplant.

[5] Nitsch D, Davenport A (2015). Designing epidemiology studies to determine the incidence and prevalence encapsulating peritoneal sclerosis (EPS). Perit Dial Int.

[6] Kawanishi H, Moriishi M, Ide K, Dohi K (2008). Recommendation of the surgical option for treatment of encapsulating peritoneal sclerosis. Perit Dial Int.

[7] Kawanishi H, Harada Y, Noriyuki T, Kawai T, Takahashi S, Moriishi M (2001). Treatment options for encapsulating peritoneal sclerosis based on progressive stage. Adv Perit Dial.

[8] Kawanishi H, Banshodani M, Yamashita M, Shintaku S, Dohi K (2019). Surgical treatment for encapsulating peritoneal sclerosis: 24 years' experience. Perit Dial Int.

[9] Margetts PJ, Bonniaud P, Liu L, Hoff CM, Holmes CJ, West-Mays JA (2005). Transient overexpression of TGF-ß1 induces epithelial mesenchymal transition in the rodent peritoneum. J Am Soc Nephrol.

[10] Khanna A, Plummer M, Bromberek C, Bresnahan B, Hariharan S (2002). Expression of TGF-beta and fibrogenic genes in transplant recipients with tacrolimus and cyclosporine nephrotoxicity. Kidney Int.

[11] Danford CJ, Lin SC, Smith MP, Wolf JL (2018). Encapsulating peritoneal sclerosis. World J Gastroenterol.

[12] Bhandari S (1996). Recovery of gastrointestinal function after renal transplantation in patients with sclerosing peritonitis secondary to continuous ambulatory peritoneal dialysis. Am J Kidney Dis.

[13] Wong CF, Beshir S, Khalil A, Pai P, Ahmad R (2005). Successful treatment of encapsulating peritoneal sclerosis with azathioprine and prednisolone. Perit Dial Int.

[14] Guest S (2009). Tamoxifen therapy for encapsulating peritoneal sclerosis: mechanism of action and update on clinical experiences. Perit Dial Int.

[15] Korte MR, Fieren MW, Sampimon DE, Lingsma HF, Weimar W, Betjes MG (2011). Tamoxifen is associated with lower mortality of encapsulating peritoneal sclerosis: results of the Dutch Multicentre EPS Study. Nephrol Dial Transplant.

[16] Dellê H, Rocha JR, Cavaglieri RC, Vieira JM, Malheiros DM, Noronha IL (2012). Antifibrotic effect of tamoxifen in a model of progressive renal disease. J Am Soc Nephrol.

[17] Farah M, Crawford RI, Levin A, Yan CC (2011). Calciphylaxis in the current era: emerging 'ironic' features?. Nephrol Dial Transplant.

[18] Schlieper G, Brandenburg V, Ketteler M, Floege J (2009). Sodium thiosulfate in the treatment of calcific uremic arteriolopathy. Nat Rev Nephrol.

[19] Mataic D, Bastani B (2006). Intraperitoneal sodium thiosulfate for the treatment of calciphylaxis. Ren Fail.

[20] Cicone JS, Petronis JB, Embert CD, Spector DA (2004). Successful treatment of calciphylaxis with intravenous sodium thiosulfate. Am J Kidney Dis.

